# Supplementary material for: Proteomic Analysis Reveals Differential Expression Profiles in Idiopathic Pulmonary Fibrosis Cell Lines
Source: Int J Mol Sci. 2022 May 1;23(9):5032. doi: 10.3390/ijms23095032 (PMC9105114; doi:10.3390/ijms23095032)
Supplement: Supplementary file 1 [file ijms-23-05032-s001.zip › Suplementary figures.pdf]

International Journal of *Molecular Sciences*

MDPI

Article

Proteomic Analysis Reveals Differential Expression Profiles in Idiopathic Pulmonary Fibrosis Cell Lines

Juan Manuel Velázquez-Enríquez [1], Alma Aurora Ramírez-Hernández [1], Luis Manuel Sánchez Navarro [2], Itayetzi Reyes-Avendaño [1], Karina González-García [1], Cristian Jiménez-Martínez [3], Luis Castro-Sánchez [4], Xariss Miryam Sánchez-Chino [5], Verónica Rocío Vásquez-Garzón [6] and Rafael Baltiérrez-Hoyos [6,*]

1 Laboratorio de Fibrosis y Cáncer, Facultad de Medicina y Cirugía, Universidad Autónoma Benito Juárez de Oaxaca, Oaxaca 68120, Mexico; juanmanuelvela_enriquez@live.com (J.M.V.-E.); aramih_09@hotmail.com (A.A.R.-H.); itayetzi.reyes94@gmail.com (I.R.-A.); k.igg@hotmail.com (K.G.-G.)

2 Facultad de Medicina y Cirugía, Universidad Autónoma "Benito Juárez" de Oaxaca, Oaxaca 68020, Mexico; sluismanuel81@hotmail.com

3 Departamento de Ingeniería Bioquímica, Escuela Nacional de Ciencias Biológicas, Instituto Politécnico Nacional, Unidad Profesional Adolfo López Mateos, Zacatenco, Av. Wilfrido Massieu Esq. Cda. Miguel Stampa S/N, Alcaldía Gustavo A. Madero, Mexico City 07738, Mexico; cjimenezh@ipn.mx

4 Conacyt-Centro Universitario de Investigaciones Biomédicas "CUIB", Universidad de Colima, Colima 28045, Mexico; luis_castro@ucol.mx

5 Catedra-Conacyt, Departamento de Salud El Colegio de La Frontera Sur, Unidad Villahermosa, Tabasco 86280, Mexico; xsanchez@ecosur.mx

6 Conacyt-Facultad de Medicina y Cirugía, Universidad Autónoma Benito Juárez de Oaxaca, Oaxaca 68120, Mexico; veronicavasgar@gmail.com

* Correspondence: rbaltierrez@hotmail.com

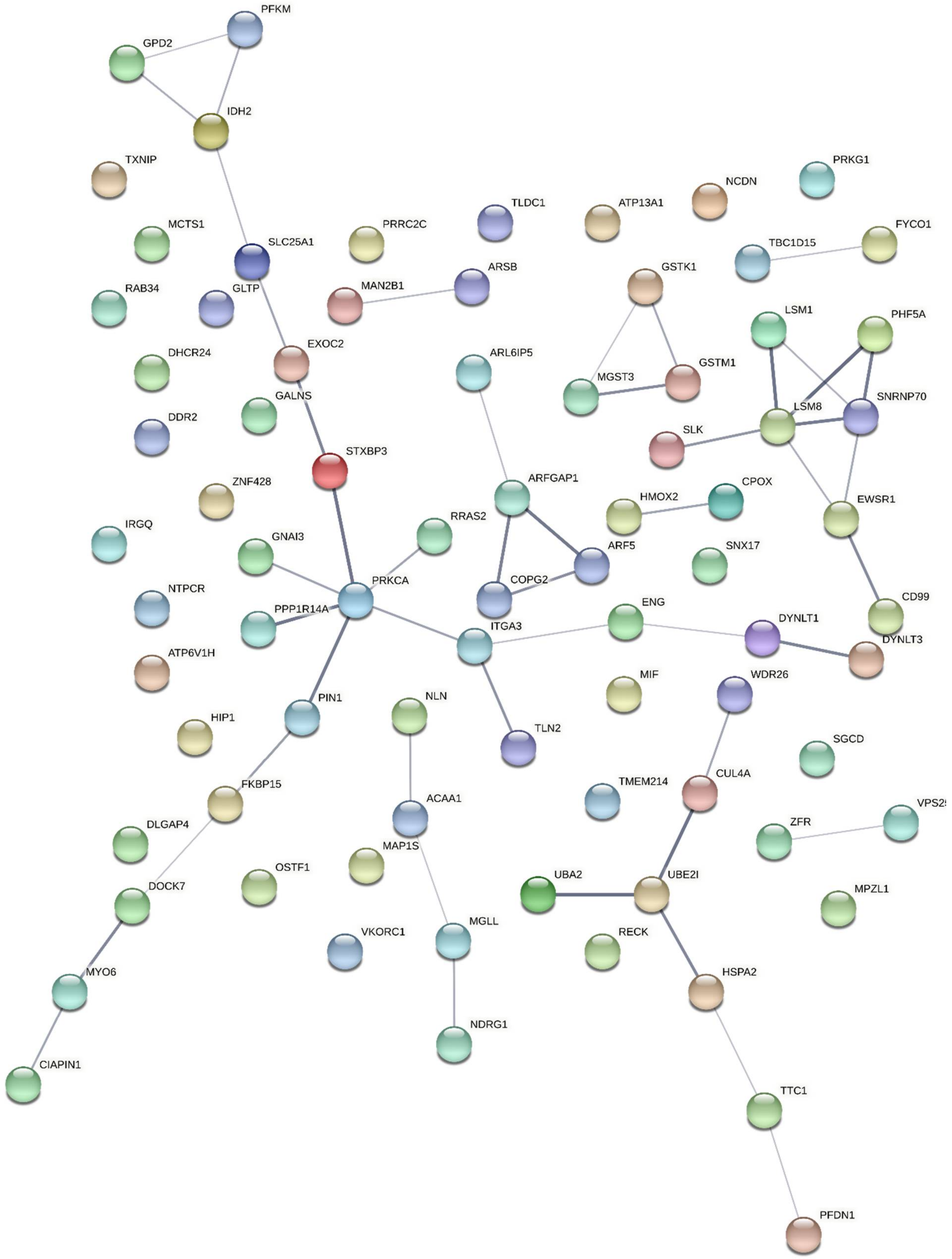

Figure S1. PPI network of the 80 proteins identified exclusively in the fibrotic groups (IPF-1 and IPF-2). The proteins were used to construct a PPI network using STRING software to visualize the interaction with tests such as network edge significance, and the active interaction sources were Text Extraction, Experiments, Database, Coexpression, Neighbourhood, Gene Fusion, and Cooccurrence, with a minimum required interaction score of medium confidence (0.4).

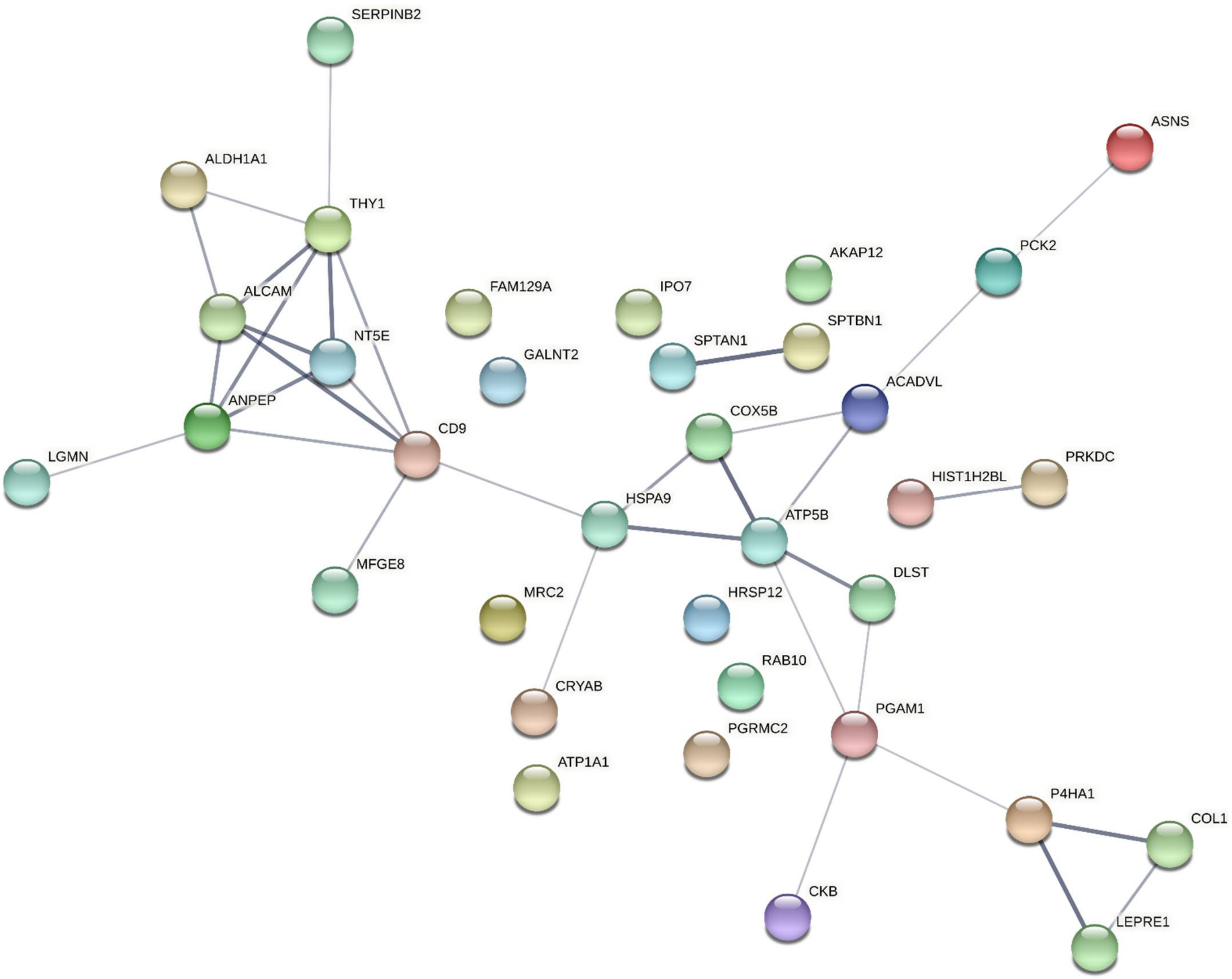

Figure S2. PPI network of DEPs in the fibrotic groups (IPF-1 and IPF-2). The proteins were used to construct a PPI network using STRING software to visualize the interaction with tests such as network edge significance and the active interaction sources were Text Extraction, Experiments, Database, Coexpression, Neighbourhood, Gene Fusion and Cooccurrence, with a minimum required interaction score of medium confidence (0.4).
